# The roles of pyroptosis in genitourinary diseases

**DOI:** 10.1007/s11255-023-03894-6

**Published:** 2023-12-16

**Authors:** Haopeng Liu, Haoran Liu, Guoshuai Huang, Hexing Yuan, Xuefeng Zhang

**Affiliations:** Department of Urology, The First Affiliated Hospital of Soochow University, 188 Shizi Road, Suzhou, China

**Keywords:** Pyroptosis, Regulated cell death, Genitourinary diseases, Mechanism, Gasdermins

## Abstract

Pyroptosis, a form of programmed cell death distinct from apoptosis and necrosis, is thought to be closely associated with the pathogenesis of diseases. Recently, the association between pyroptosis and urinary diseases has attracted considerable attention, and a comprehensive review focusing on this issue is not available. In this study, we reviewed the role of pyroptosis in the development and progression of benign urinary diseases and urinary malignancies. Based on this, pyroptosis has been implicated in the development of urinary diseases. In summary, this review sheds light on future research directions and provides novel ideas for using pyroptosis as a powerful tool to fight urinary diseases.

## Introduction

Pyroptosis is a newly discovered form of programmed cell death. Cell death is usually categorized as nonprogrammed cell death and programmed cell death (PCD) [1]. Pyroptosis is a type of inflammatory PCD [2]. The process of pyroptosis was first described in 1992, but the term was coined in 2001 following the observation that bacterium-infected macrophages underwent rapid lytic cell death dependent on caspase-1 activity [3]. Recently, macrophages were shown to regulate pyroptosis and play an important role in the development of acute kidney injury (AKI), diabetic nephropathy (DN) and renal fibrosis [4–6]. Pyroptosis is characterized by cell membrane pore formation, cell swelling, and the release of inflammatory intracellular contents [7, 8]. The inflammatory factors released during cell lysis, such as interleukin-1β (IL-1β) and interleukin-18 (IL-18), amplify the inflammatory effects and activate immune responses [7, 8].

The underlying mechanism was only uncovered upon the discovery of gasdermin D (GSDMD) protein. Shi et al. found that caspase-1/11/4/5 can induce pyroptosis by cleaving GSDMD to release its N-terminal domain [9]. In addition to GSDMD, the gasdermin family also includes five other members. The human gasdermin family comprises GSDMA, GSDMB, GSDMC, GSDMD, GSDME/DFNA5, and PVJK/DFNB59. In mice, there are five gasdermin members, namely, GSDMA, GSDMC, GSDMD, GSDME, and PJVK/DFNB59, but not GSDMB [2]. All gasdermins except DFNB59 have two conserved domains: an N-terminal effector domain and a C-terminal inhibitory domain [2].

Normally, moderate pyroptosis contributes to host defence against pathogen infection, but excessive pyroptosis leads to uncontrolled inflammatory responses, massive cell death, and serious tissue damage, causing inflammatory or autoimmune diseases [2]. As a proinflammatory type of cell death, pyroptosis provides a new opportunity for cancer elimination by activating the anti-tumour immune response [2]. An increasing number of studies have shown that pyroptosis plays a crucial role in many cancers, such as breast cancer, gastric cancer, and lung cancer [10–12].

Here, we first describe the different signalling pathways of pyroptosis to gain an in-depth understanding of the molecular mechanism. Finally, the role of pyroptosis in urinary diseases is discussed, followed by suggestions for future research directions.

## Overview of pyroptosis

### Canonical pathway

**Fig. 1:**
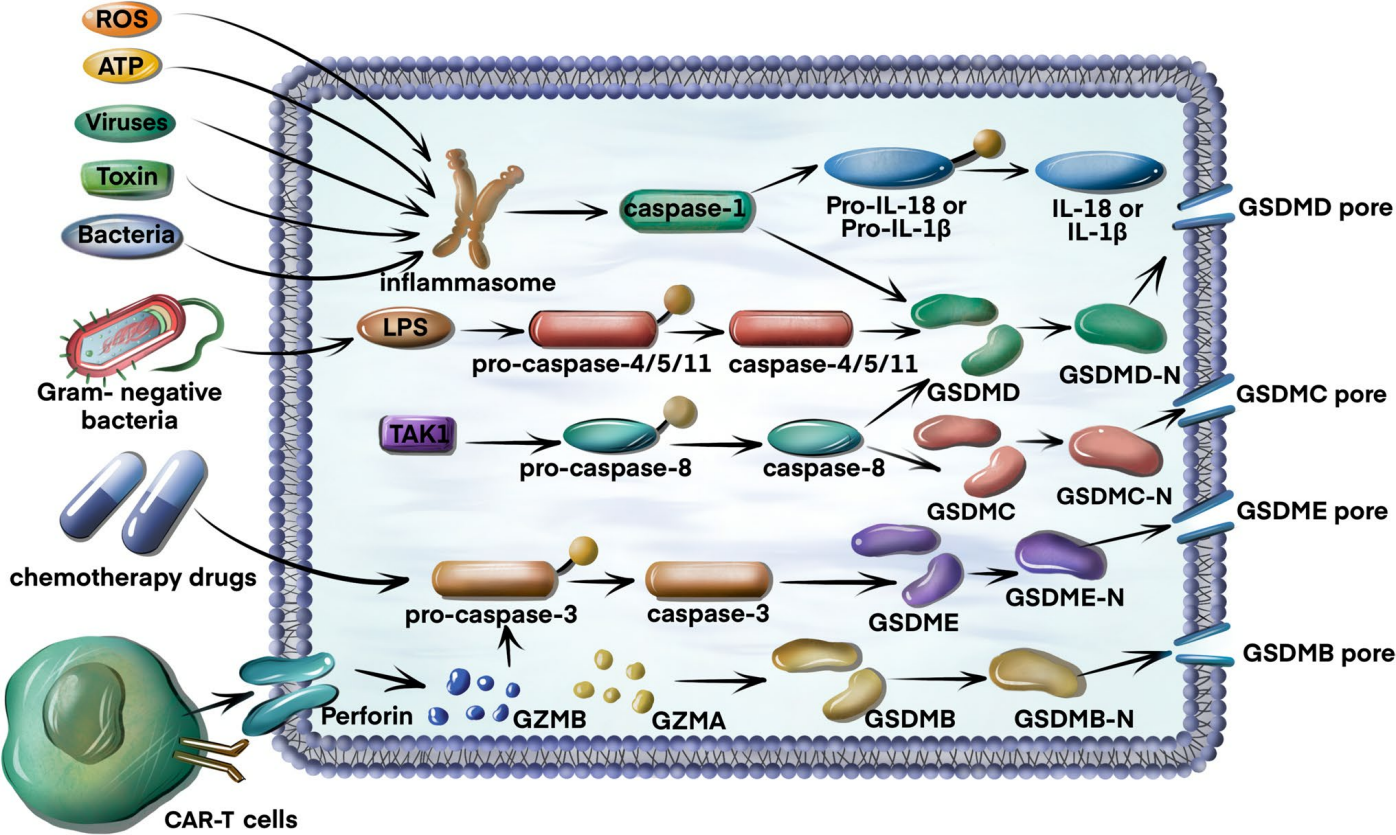
Molecular mechanisms of the canonical pathway, non-canonical pathway, apoptotic caspase-mediated pathway and granzyme-mediated pathway in pyroptosis. In the canonical pathway, pathogen-associated molecular patterns or damage-associated molecular patterns (such as ROS, ATP, viruses, bacteria, or toxins) stimulate inflammasomes, which then activate caspase-1. Activated caspase-1 not only cleaves inactive IL-1β and IL-18 precursors but also cleaves GSDMD, which forms pores and induces pyroptosis. In the non-canonical pathway, LPS from Gram-negative bacteria activates caspase-4/5/11, and activated caspase-4/5/11 cleaves GSDMD to promote pyroptosis. In the apoptotic caspase-mediated pathway, caspase-3/GSDME, caspase-8/GSDMD and caspase-8/GSDMC mechanisms can promote pyroptosis. In the granzyme-mediated pathway, GZMA or GZMB derived from CAR-T cells cleaves GSDMB or GSDME, respectively, to induce pyroptosis. *ASC* caspase-recruitment domain, *ROS* reactive oxygen species, *IL-18* interleukin-18, *IL-1β* interleukin-1β, *LPS* lipopolysaccharide, *TAKI* TGF-β-activated kinase-1, *GSDMD* gasdermin D, *GSDME* gasdermin E, *GSDMB* gasdermin B, *GZMB* granzyme B, *GZMA* granzyme A

The classical pyroptosis pathway is mediated by caspase-1 [13]. Inflammasomes are formed by pattern-recognition receptors (PRRs, also known as inflammasome sensors), apoptosis-associated speck-like protein containing a caspase-recruitment domain (ASC), and inactive pro-caspase-1 [13–15]. PRRs can recognize pathogen-associated molecular patterns and danger-associated molecular patterns (PAMPs and DAMPs) [16, 17]. PRRs include nucleotide-binding oligomerization domain-like receptors (NLRs, including NLRP1, NLRP3, and NLRC4), absent in melanoma 2 (AIM2), and pyrin [18, 19]. NLRs usually consist of a leucine-rich repeat (LRR), a nucleotide-binding oligomerization domain (NACHT/NOD), and a caspase-recruitment domain (CARD) or pyrin domain (PYD) and are divided into NLRPs or NLRCs according to whether their N-terminus contains a PYD or CARD [20]. A PYD is needed for interaction with ASC. The NOD participates in adenosine triphosphate (ATP)-dependent activation of the signal. The LRR is responsible for ligand recognition and autoinhibition. The CARD participates in pro-caspase-1 recruitment [2]. Upon receiving an activating signal, inflammasome sensors recruit pro-caspase-1 (which has a CARD) either directly through homotypic binding of CARD or indirectly through the PYD by means of ASC, which contains a PYD and a CARD [17]. Subsequently, caspase-1 activation occurs through self-cleavage. Activated caspase-1 not only cleaves inactive IL-1β and IL-18 precursors but also cleaves GSDMD to form GSDMD-NT and GSDMD-CT [21–24]. GSDMD-N forms pores in the plasma membrane, leading to cell swelling and pyroptosis [25, 26] (Fig. 1).

### Non-canonical pathway

Most gram-negative bacteria activate the non-canonical inflammasome pathway [2]. The nonclassical signalling pathway is mediated by caspase-4 and caspase-5 in humans and by caspase-11 in mice [27, 28]. These caspases can be activated by directly binding to lipopolysaccharide (LPS) [28]. Activated caspase-4/5/11 cleaves GSDMD to promote pyroptosis. However, caspase-4/5/11 cannot cleave pro-IL-18/pro-IL-1β but can cleave GSDMD, which can cause K^+^ efflux and NLRP3/caspase-1 pathway activation, eventually leading to the maturation and release of interleukin-18 (IL-18) and interleukin-1β (IL-1β) [9, 14, 29] (Fig. 1).

### Apoptotic caspase-mediated pathway

In addition to inflammatory caspase-1/4/5/11, some apoptotic caspases can also trigger pyroptosis. Chemotherapeutic drugs can induce caspase-3 to cleave GSDME to form GSDME-N termini, which cause pyroptosis [30, 31]. In addition, pathogenic Yersinia has been shown to inhibit TGFβ-activated kinase-1 (TAK1) via the Yersinia effector protein YopJ and induce caspase-8-related cleavage of GSDMD to elicit pyroptosis [32, 33]. Interestingly, caspase-8 induces GSDMC cleavage, thereby leading to a non-canonical pyroptosis pathway in cancer cells [34] (Fig. 1).

### Granzyme-mediated pathway

Granzyme A (GzmA) is the most abundant serine protease of the granzyme family and has traditionally been recognized as a mediator of cell death [2]. Zhou et al. found that GZMA derived from cytotoxic T lymphocytes cleaves GSDMB to induce pyroptosis [35]. In 2020, it was reported that CAR-T cells activated caspase-3 by releasing granzyme B (GzmB), subsequently leading to the activation of the caspase-3/GSDME-mediated pyroptotic pathway, thus causing pyroptosis [36]. Additionally, Zhang et al. found that GzmB directly cleaved GSDME and induced pyroptosis, enhancing anti-tumour immunity and inhibiting tumour growth [37] (Fig. 1).

## Pyroptosis in benign urinary diseases

### Pyroptosis in interstitial cystitis

Interstitial cystitis (IC), also known as bladder pain syndrome (BPS), is a chronic pain disorder that most commonly presents in the bladder, pelvis, or abdomen [38]. Pyroptosis plays an important role in the development of IC. A study showed that the NLRP3 inflammasome is a crucial player in the development of bladder disease [39]. Some results have demonstrated that the expression levels of NLRP3, caspase-1, and GSDMD in patients with IC are elevated [40, 41]. Wang et al. found that the NLRP3/GSDMD-N pathway was activated and played a role in the development of IC [42]. Wang et al. showed that aster tataricus extract (ATE) can be used as an inhibitor of NLRP3 in treating IC [43]. The discovery of NLRP3/caspase-1/GSDMD-N as a new pathway provides a new direction for IC research.

### Pyroptosis in BPH

Benign prostatic hyperplasia (BPH) is characterized by the nonmalignant overgrowth of prostatic tissue surrounding the urethra, ultimately constricting the urethral opening and giving rise to associated lower urinary tract symptoms (LUTS) such as urgency, frequency, nocturia, incomplete bladder emptying, and a weak urine stream [44]. There is much evidence to suggest that inflammation plays an important role in BPH. It has been reported that the expression levels of NLRP1 and caspase-1, IL-18 and IL-1β are elevated in BPH [45]. Therefore, the NLRP1/caspase-1 pathway is activated and participates in the development of BPH. Jiang et al. found that peroxiredoxin 3 (PRDX3) suppressed autophagy flux and activated pyroptosis to induce inflammatory responses and stimulate the overgrowth of prostate tissues [46]. Emerging results indicate that steady-state levels of AIM2 mRNA are higher in BPH tissue than in normal prostate tissue [47]. AIM2 recruits ASC and pro-caspase-1 to assemble the AIM2 inflammasome, leading to cell swelling and pyroptosis. These studies have facilitated the identification of potential BPH treatment targets. The signalling pathways regulating pyroptosis in BPH are displayed in Fig. 3.

### Pyroptosis in AKI

**Fig. 2:**
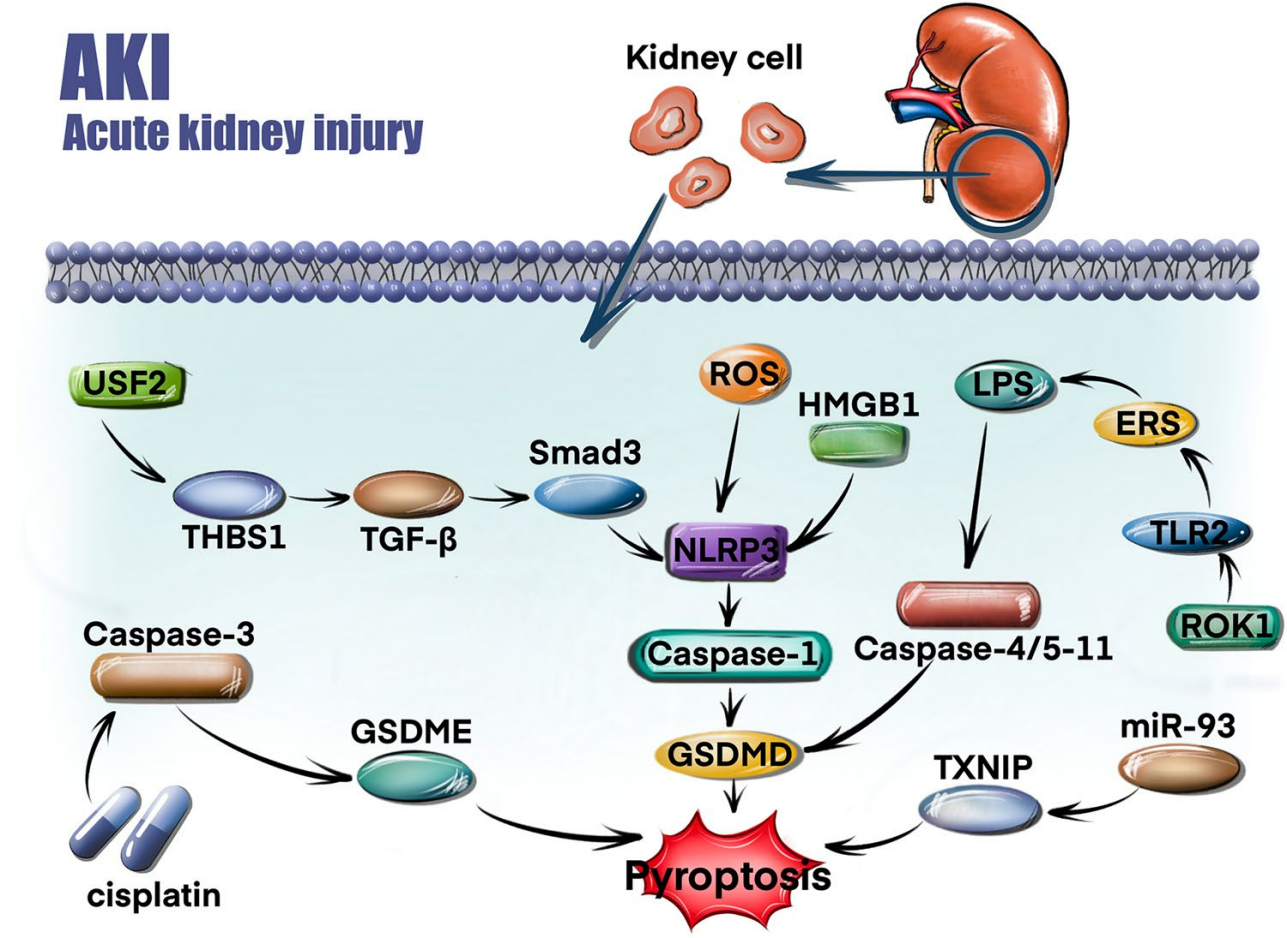
Signalling pathways regulating pyroptosis in AKI. THBS1 is upregulated by USF2 and activates the TGF-β/Smad3/NLRP3/caspase-1 signalling pathway, thus inducing pyroptosis. NLRP3 is upregulated by HMGB1 and activates the expression of GSDMD. ROS induce pyroptosis via the NLRP3/caspase-1/GSDMD signalling axis. Cisplatin induces pyroptosis via the caspase-3/GSDME signal axis. miR-93 targets TXN2P and thus induces pyroptosis. ROCK1 regulates LPS-induced pyroptosis via TLR2-mediated ERS. *USF2* upstream stimulatory factor 2, *ROS* reactive oxygen species, *THBS1* thrombospondin-1, *TGF-β* transforming growth factor-β, *NLRP3* NOD-like receptor 3, *HMGB1* high mobility group box 1, *LPS* lipopolysaccharide, *ERS* endoplasmic reticulum stress, *TLR2* toll-like receptor 2, *ROCK1* Rho-associated coiled-coil containing protein kinase-1, *TXNIP* thioredoxin–interacting protein, *miR-93* microRNA-93, *GSDMD* gasdermin D, *GSDME* gasdermin E

Acute kidney injury (AKI) is defined by a rapid increase in serum creatinine, a decrease in urine output, or both [48]. Recent advances have revealed a role for pyroptosis in AKI. Sun et al. found that thrombospondin-1 (THBS1) and upstream stimulatory factor 2 (USF2) were highly expressed in patients with sepsis-induced AKI and that USF2 upregulated THBS1 expression to activate the TGF-β/Smad3/NLRP3/caspase-1 signalling pathway and stimulate pyroptosis, ultimately exacerbating sepsis-induced AKI [49]. Miao et al. found that the expression of GSDMD was significantly increased in both cisplatin-induced and ischaemia‒reperfusion (I/R) models [50]. The knockout of caspase-11 or GSDMD alleviated kidney damage in mice with cisplatin-induced AKI. A study published in 2020 showed that the protein levels of high mobility group box 1 (HMGB1), IL-1β, IL-18, NLRP3, and GSDMD were elevated in an AKI model [6]. Therefore, we hypothesize that the HMGB1/NLRP3/GSDMD signalling pathway plays a pivotal role in the pathogenesis of AKI. In addition, Li et al. demonstrated that the ROS/NLRP3/caspase-1/GSDMD pathway mediated contrast-induced AKI (CI-AKI) via pyroptosis and that baicalin treatment alleviated the associated inflammation and oxidation levels [51]. Studies have also shown that macrophage-derived exosomal miRNAs play important roles in AKI [52, 53]. Xia et al. found that the levels of GSDME-N and IL-1β were elevated in cisplatin-induced AKI [54]. The inhibition of caspase-3 blocked GSDME-N cleavage and attenuated cisplatin-induced pyroptosis and kidney dysfunction. Therefore, caspase-3/GSDME-triggered pyroptosis plays an important role in AKI. Juan et al. found that the exosomal miR-93/thioredoxin–interacting protein (TXNIP) signalling pathway plays a crucial role in the progression of sepsis-induced AKI and that M1 exosomes promote pyroptosis and M2 exosomes inhibit pyroptosis [55]. It has been well established that Rho-associated coiled-coil containing protein kinase-1 (ROCK1) plays an important role in a series of pathological processes, including pyroptosis, inflammation, and endoplasmic reticulum stress (ERS) [56, 57]. Wang et al. found that ROCK1 regulates LPS-induced kidney cell pyroptosis via Toll-like receptor 2 (TLR2)-mediated ERS, thereby accelerating sepsis-induced AKI progression [58]. The signalling pathways regulating pyroptosis in AKI are displayed in Fig. 2.

### Pyroptosis in DN

Diabetic nephropathy (DN), or diabetic kidney disease (DKD), is a frequent and severe long-term microvascular complication resulting from lesions in the renal glomeruli and tubules [59]. Growing evidence has demonstrated that chronic inflammation promotes the pathogenesis of DN [60]. The role of pyroptosis signalling pathways in DN progression has attracted the attention of researchers and clinicians. In 2020, it was reported that the TXNIP/NLRP3 axis is an important pathway that regulates DN induced by pyroptosis [61]. Interestingly, Ke et al. found that the ERS-related factor IRE1α upregulated TXNIP/NLRP3 inflammasome-induced pyroptosis in DN rats [62]. Li et al. found that NLRP3/caspase-1/GSDMD signalling was strikingly upregulated and the secretion of IL-1β and IL-18 dramatically increased in DN mice [63]; in addition, they also confirmed that SYR inhibited the NLRP3/caspase-1/GSDMD pyroptosis pathway by upregulating NRF2 signalling in DN. Li et al. found that the expression of p-NF-κB, ASC, cleaved-IL-1β, NLRP3, cleaved-caspase-1, and GSDMD-N was elevated in a DN mouse model [64]; in addition, they confirmed that geniposide (GE) may inhibit the development of DN via the APMK/SIRT1/NF-κB pathway [64]. The APMK/SIRT1/NF-κB axis may become a new signalling pathway for the treatment of DN. In addition, NLRP3 inflammasome activation is related to the pathogenesis of DN. Wang et al. revealed that the expression of NLRC4, IL‑1β, and IL‑18 was increased under high glucose conditions, inducing pyroptosis in renal tubular epithelial cells [65]. Komada et al. demonstrated that the activation of the AIM2 inflammasome by DNA from necrotic cells drives pyroptosis, which contributes to chronic kidney injury [66]. Cheng et al. demonstrated that caspase-11/4- and GSDMD-mediated pyroptosis was activated in a DN mouse model and involved in the development of DN [67]. In summary, these findings confirm that pyroptosis and inflammasomes play important roles in renal injury, ultimately affecting the pathogenesis of DN.

## Pyroptosis in urinary malignancies

### Pyroptosis in bladder cancer

Bladder cancer (BCa) is the most common malignancy of the urinary tract [68]. Recent advances have revealed an important role of pyroptosis in bladder cancer. He et al. found that GSDMB binds to signal transducer and activator of transcription 3 (STAT3) and increases the phosphorylation of STAT3, which increases the expression of hexokinase 2 (HK2), lactate dehydrogenase A (LDHA), enolase 2 (ENO2), and insulin-like growth factor-binding protein 3 (IGFBP3) to enhance glycolysis in BCa cells and promote cancer cell proliferation [69]; in addition, they also demonstrated that ubiquitin-specific peptidase 24 (USP24) interacts with GSDMB and prevents GSDMB degradation in BCa cells [69]. Therefore, the USP24/GSDMB/STAT3 axis may become a new targetable signalling pathway for bladder cancer treatment. Chen et al. showed, based on K‒M curves, that GSDMB and CASP6 are associated with better prognoses for patients with BCa [70]; they also found that many tumours with high GSDMB and CASP6 expression were immune-inflamed tumours and that many tumours with low GSDMB and CASP6 expression were immune-desert tumours. Then, they demonstrated that GSDMB and CASP6 play important roles in immune infiltration [70]. The results from El-Gamal et al. showed that the expression level of GSDMD in muscle-invasive bladder cancer (MIBC) was significantly higher than that in non-muscle-invasive bladder cancer (NMIBC) and that the expression level in NMIBC was higher than that in the control group [71]. These results show that GSDMD is involved in the pathogenesis of BCa and muscle invasion. In addition, the expression of GSDMD in tissue can be used as a useful tool for predicting local tumour recurrence [71]. Peng et al. found that CD147 promoted cell proliferation in BCa by upregulating the expression of GSDMD [72].

### Pyroptosis in prostate cancer

Prostate cancer (PCa) is a major disease that affects men’s health worldwide. It is the second most common form of cancer in men, surpassed only by nonmelanoma skin cancers such as basal and squamous cell carcinomas [73]. Pyroptosis is also involved in PCa development. As a classical pyroptosis pathway, the caspase-1 pathway plays an important role in PCa. NLRP3 participates in physiological and pathological processes, including tumour progression. In 2021, Xu et al. found that the expression of NLRP3 in PCa tissues and cell lines was elevated and was positively correlated with that of caspase-1 [74]. Their results revealed that the NLRP3 inflammasome exerted a tumour-promoting effect by activating caspase-1 in PCa [74]. Karan et al. reported that the expression of NLRP12 was significantly higher in PCa tissue than in adjacent benign tissue and that NLRP12 may play an important role in activating NF-κB and IL-1β signalling and its association with the pathogenesis and progression of PCa [75]; they indicated that NLRP12 can upregulate caspase-1, IL-1 β, and IL-18 to promote the occurrence and progression of PCa. Many studies have shown that LPS participates in the proliferation, migration, and invasion of PCa cells [76–78]. It has been shown that LPS activates the caspase-4/5/11 pathway to induce pyroptosis [28]. However, LPS-mediated pyroptosis is still being investigated in PCa.

### Pyroptosis in renal cell carcinoma

Renal cell carcinoma (RCC) accounts for 2–3% of all malignant diseases in adults [79]. It is the seventh most common cancer in men and the ninth most common in women [79]. The most common RCC is clear cell RCC (ccRCC) (70–90%), followed by papillary RCC (10–15%) and chromophobe RCC (3–5%) [80]. In recent years, researchers have found that pyroptosis is inextricably linked to the development of RCC. Cui et al. found that GSDMB expression was significantly more upregulated in ccRCC tissues than in surrounding normal tissues [81]; in addition, they confirmed that the upregulation of GSDMB is significantly related to immune infiltrates and poor survival in ccRCC [81]. GSDMB has the potential to become a biomarker for poor prognosis and a potential target for immune therapy in ccRCC. Liver X receptors [LXRs; nuclear receptor subfamily 1, group H, member 2 (NR1H2, also known as LXRB) and nuclear receptor subfamily 1, group H, member 3 (NR1H3, also known as LXRA)] belong to the nuclear receptor superfamily and are expressed in various cells [82]. Wang et al. found that the expression levels of NLRP3 in ccRCC tissue were significantly lower than those in normal kidney tissue and that LXRα promoted tumour metastasis by downregulating the NLRP3 inflammasome in ccRCC [83]. In addition, bromodomain-containing 4 (BRD4) inhibition was shown to prevent cell proliferation and epithelial–mesenchymal transition (EMT) and play an anti-tumour role in RCC by activating the NF-κB–NLRP3–caspase-1 pyroptosis signalling pathway [84]. Zhang et al. found that the expression of most pyroptosis regulatory genes is positively correlated and plays an important prognostic role in ccRCC [85]. AIM2 plays a crucial role in the development of various tumours. Recent studies have shown that AIM2 is highly expressed in ccRCC and promotes tumour development through immune activation pathways [86]. Tang et al. found that lncRNA FOXD2 adjacent opposite strand RNA 1 (FOXD2-AS1) affects GSDMB and NLRP1 [87]; interestingly, they also found that downregulating the expression of FOXD2-AS1 reduced the proliferation and migration of ccRCC cells [87]. This indicates that FOXD2-AS1 may provide a new direction for research on the treatment of RCC.

## Conclusion

**Fig. 3:**
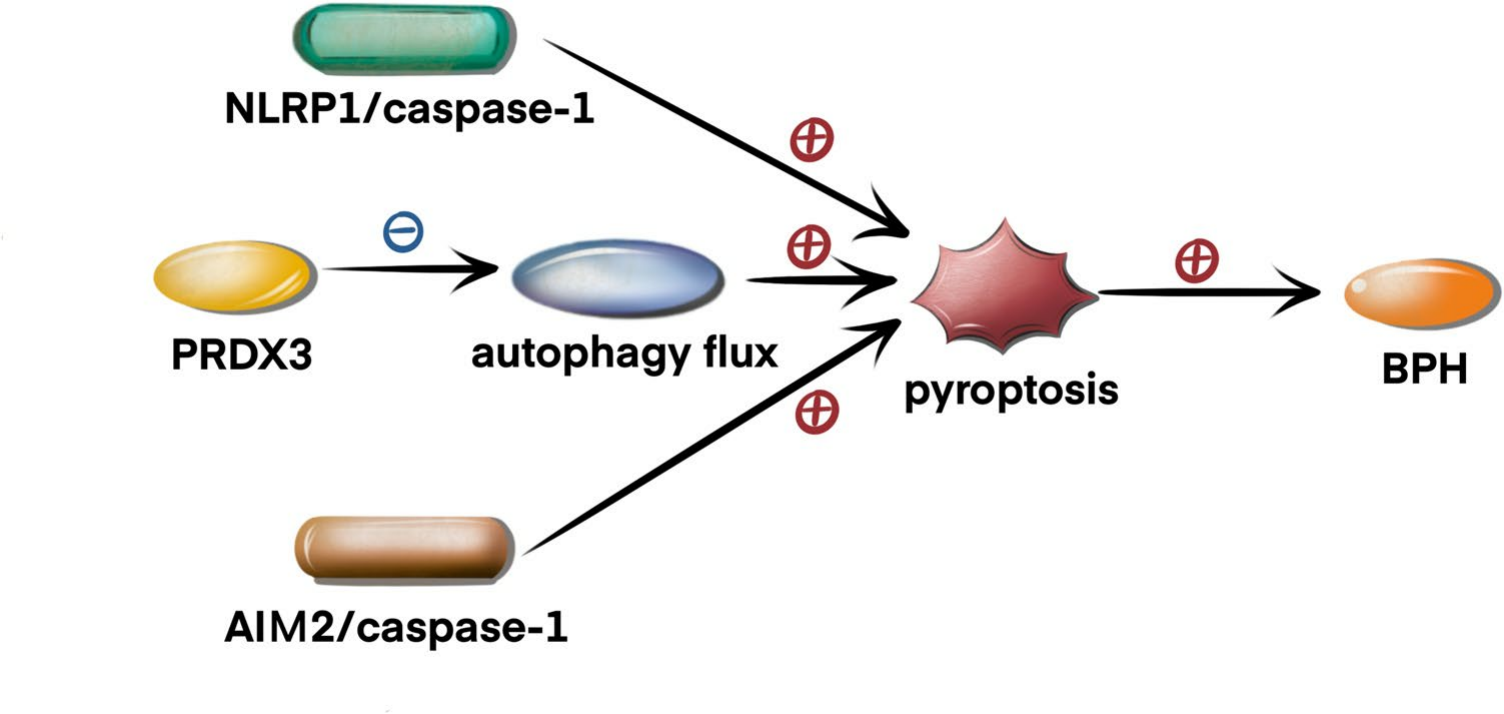
Signalling pathways regulating pyroptosis in BPH. NLRP1/caspase-1 induces pyroptosis to promote the development of BPH. PRDX3 suppresses autophagy flux and activates pyroptosis to promote the development of BPH. AIM2/caspase-1 induces pyroptosis to promote the development of BPH. *PRDX3* peroxiredoxin 3, *NLRP1* NOD-like receptor 1, *AIM2* absent in melanoma 2, *BPH* benign prostatic hyperplasia

In conclusion, pyroptosis is a newly identified form of cell death mediated by gasdermin proteins, which are often activated by caspases. It plays a crucial role in the occurrence, development, and progression of urologic diseases. The molecular mechanism of pyroptosis is shown in Fig. 1. The signalling pathways regulating pyroptosis in AKI are shown in Fig. 2. The signalling pathways regulating pyroptosis in BPH are shown in Fig. 3. Future in-depth research on pyroptosis in urological diseases will help us better understand the diagnosis and treatment of urinary diseases. Future studies are urgently needed to develop more clinical trials to explore the potential application of pyroptosis in urinary diseases.

## Abbreviations

ASC: Caspase-recruitment domain
ROS: Reactive oxygen species
IL-18: Interleukin-18
IL-1β: Interleukin-1β
LPS: Lipopolysaccharide
TAK1: TGF-β-activated kinase-1
GSDMA: Gasdermin A
GSDMB: Gasdermin B
GSDMC: Gasdermin C
GSDMD: Gasdermin D
GSDME: Gasdermin E
GZMB: Granzyme B
GZMA: Granzyme A
USF2: Upstream stimulatory factor 2
THBS1: Thrombospondin-1
NLRP1: NOD-like receptor 1
AIM2: Absent in melanoma 2
NLRP3: NOD-like receptor 3
HMGB1: High mobility group box 1
ERS: Endoplasmic reticulum stress
TLR2: Toll-like receptor 2
ROCK1: Rho-associated coiled-coil containing protein kinase-1
TXNIP: Thioredoxin–interacting protein
miR-93: MicroRNA-93
PRDX3: Peroxiredoxin 3
STAT3: Transcription 3
HK2: Hexokinase 2
LDHA: Lactate dehydrogenase A
ENO2: Enolase 2
USP24: Ubiquitin-specific peptidase 24
IGFBP3: Insulin-like growth factor-binding protein 3
